# The inflammation–epigenetics axis in gastric cancer: Helicobacter-driven field defects, immune escape, and therapeutic frontiers

**DOI:** 10.3389/fimmu.2026.1955428

**Published:** 2026-09-16

**Authors:** Yue Bai, Zhongran Lu

**Affiliations:** 1 Combined Ward of Traditional Chinese and Western Medicine, Shengli Oilfield Central Hospital, Dongying, Shandong, China; 2 Department of Gastroenterology, Shengli Oilfield Central Hospital, Dongying, Shandong, China

**Keywords:** chronic gastritis, DNA methylation, epigenetic field cancerization, Epstein–Barr virus, gastric cancer, *Helicobacter pylori*, immunotherapy, liquid biopsy

## Abstract

Gastric cancer provides a clinically relevant model of the inflammation–epigenetics axis because Helicobacter pylori can drive prolonged mucosal inflammation before malignant transformation. Inflammatory signaling remodels DNA methylation, histone states, chromatin accessibility and RNA-regulatory programs, generating epigenetic field defects that may persist after bacterial eradication. Epstein–Barr virus-positive gastric cancer represents a distinct hypermethylated and immune-adapted state. We organize these processes using four coordinates: inflammatory driver, dominant epigenetic lesion, cellular compartment and reversibility. Across gastric cancer subtypes, epigenetic plasticity contributes to field cancerization, antigen-presentation loss, epithelial–mesenchymal transition, stemness and drug-tolerant persistence, while parallel reprogramming of immune and stromal compartments promotes treatment resistance. Tissue and circulating DNA methylation currently have stronger translational support than circulating non-coding RNA or m6A-based signatures, which remain investigational because of source attribution, assay standardization and validation limitations. Therapeutic translation should therefore combine removal of the sustaining inflammatory driver with biomarker-guided epigenetic priming and state-matched immune or stromal intervention. Longitudinal and externally validated studies are required to convert these mechanisms into clinically useful biomarkers and treatment strategies.

## 1 Introduction

Gastric carcinogenesis unfolds within a tissue that is continuously exposed to microbes, dietary compounds, acid, bile, mechanical damage and immune surveillance. This makes inflammation both a necessary component of mucosal defense and a long-term carcinogenic pressure. The Correa cascade—from chronic active gastritis through atrophy, intestinal metaplasia and dysplasia—captures the histological sequence of intestinal-type cancer, but it does not fully explain why only a subset of exposed individuals progress. Epigenetic field cancerization supplies a mechanistic bridge: repeated inflammation leaves durable molecular memory across non-neoplastic glands, creating a mucosal substrate in which oncogenic clones are more likely to emerge and persist (1–6).

The most compelling human evidence comes from H. pylori-associated methylation. Gastric mucosa from infected individuals shows markedly elevated promoter methylation at multiple loci; experimental work indicates that host inflammation, rather than direct bacterial contact alone, is the principal inducer. Eradication reduces active inflammation and may lower methylation at selected loci, yet residual methylation can persist for years and stratify future cancer risk (3–8). This persistence is clinically important because bacterial status and histology no longer fully capture the biological memory of prior exposure.

A second route is Epstein–Barr virus (EBV)-associated gastric cancer, in which latent viral infection, immune stimulation and viral control of host chromatin produce one of the most extensive CpG island hypermethylation phenotypes observed in human tumors. The Cancer Genome Atlas (TCGA) therefore positioned gastric cancer into four molecular classes—Epstein–Barr virus (EBV)-positive, microsatellite instability (MSI), genomically stable (GS), and chromosomal instability (CIN)—each with a distinct mixture of inflammatory inputs, genetic competence and epigenetic architecture (9). The inflammation–epigenetics axis is consequently not one pathway but a family of state transitions that converge on immune escape, plasticity and treatment resistance.

This review evaluates the evidence linking inflammatory exposure to epigenetic remodeling, persistent epithelial and microenvironmental states, clinical phenotype, and therapeutic response in gastric cancer. Emphasis is placed on causal evidence, cell-type specificity, reversibility, and clinical validation rather than on cataloguing individual enzymes or methylated loci.

### 1.1 Review scope and conceptual framework

Four coordinates are used throughout. First, what is the sustaining inflammatory driver—active H. pylori infection, residual post-eradication inflammation, EBV latency, autoimmune atrophy, bile reflux, obesity-associated metabolic stress or treatment-induced wound repair? Second, what is the dominant epigenetic lesion—DNA methylation, loss of 5-hydroxymethylcytosine, repressive histone marks, enhancer addiction, non-coding RNA circuitry or RNA modification? Third, which compartment carries that lesion—epithelial field, malignant clone, myeloid cell, lymphocyte, fibroblast or endothelium? Fourth, is the state reversible, and over what time scale?

Mechanistic interpretation requires four variables to be resolved: the inflammatory driver, the dominant epigenetic lesion, the cell population carrying that lesion, and its reversibility. Bulk-tissue measurements cannot reliably distinguish malignant epithelium from metaplastic glands, infiltrating immune cells, or stroma; spatial, single-cell, and longitudinal analyses are therefore necessary for causal attribution (10–22).

### 1.2 Evidence scope and interpretation

The review emphasizes mechanistic human studies, infection and inflammation models, molecular classifications, prospective biomarker cohorts, randomized eradication trials and gastric-cancer-specific translational studies. Evidence from other tumors is included only where it defines a conserved epigenetic or immune mechanism relevant to gastric cancer.

## 2 Gastric inflammatory ecosystems as epigenetic selection pressure

### 2.1 *H. pylori*, CagA and chronic active gastritis

H. pylori establishes a long-lived niche in the gastric mucosa and activates innate and adaptive immunity through pattern-recognition receptors, epithelial stress and bacterial virulence factors. CagA-positive strains inject CagA into epithelial cells, perturbing polarity, junctional signaling, SHP2, β-catenin and inflammatory transcription. Neutrophils and macrophages generate reactive oxygen species (ROS) and reactive nitrogen species, while epithelial, stromal and immune cells produce IL-1β, TNF-α, IL-6, IL-8 and prostaglandins. NF-κB and AP-1 drive acute inflammatory genes; STAT3 supports survival and regenerative proliferation; TGF-β and hypoxia progressively favor metaplastic, fibrotic and immune-excluded states (2–5, 23, 24).

Inflammation-dependent methylation is a key causal link. In gerbil and mouse models, suppression of inflammation substantially attenuates aberrant methylation despite continued exposure to bacterial components, whereas macrophage-derived nitric oxide can promote methylation and silencing of genes such as RUNX3. IL-1β-dependent inflammatory signaling also contributes to methylation accumulation. These findings argue that the critical carcinogenic unit is the infected mucosal ecosystem rather than the bacterium in isolation (4, 24).

The field effect is heterogeneous. Stem and progenitor cells within inflamed glands may retain methylation errors during clonal expansion; differentiated cells can display transient changes that disappear with epithelial turnover. Atrophy and metaplasia alter the cellular composition of biopsies and can themselves stabilize lineage-specific enhancer states. Consequently, the same methylation measurement can represent a reversible inflammatory response, a durable stem-cell lesion or a shift in cell mixture. Longitudinal sampling and cell-type deconvolution are essential.

### 2.2 EBV, autoimmune atrophy, bile reflux and metabolic inflammation

EBV-positive gastric cancer represents a distinct viral inflammatory–epigenetic archetype. Viral latency proteins and non-coding RNAs interact with host chromatin and DNA methylation machinery, while the immune system exerts sustained selection on infected epithelial clones. EBV-positive tumors exhibit extreme promoter hypermethylation, recurrent PIK3CA mutation, frequent ARID1A alteration and enrichment of JAK2/PD-L1/PD-L2 amplification or expression. Unlike MSI-associated CpG island methylator phenotype (CIMP), EBV-CIMP typically includes CDKN2A methylation but not the MLH1 methylation that defines many sporadic MSI tumors (9, 25–28).

Autoimmune gastritis creates a non-infectious route to chronic atrophy, hypergastrinemia and altered epithelial differentiation. Bile reflux and post-surgical or cardia inflammation expose the mucosa to detergents, oxidative stress and NF-κB activation. Obesity and insulin resistance add systemic IL-6, leptin, altered lipid flux and hypoxia. These drivers differ in topography and cellular targets but converge on regenerative signaling, metabolic control of epigenetic cofactors and selection of stress-tolerant clones.

This convergence does not erase etiological specificity. Viral latency can directly maintain a tumor-cell methylome; H. pylori creates a broad mucosal field; autoimmune injury preferentially remodels the corpus; and bile-associated injury affects junctional or remnant stomach contexts. Biomarkers and interventions should therefore preserve information about anatomical site, histological background and current inflammatory activity.

### 2.3 Metabolic and treatment-induced inflammation

Inflammation changes the availability of epigenetic substrates. S-adenosylmethionine supports DNA and histone methylation; acetyl-CoA supports histone acetylation; α-ketoglutarate is required by ten-eleven translocation (TET) enzymes and Jumonji-domain histone demethylases; NAD+ regulates sirtuins. Hypoxia, lactate, succinate, lipid accumulation and one-carbon imbalance can therefore alter chromatin without changing enzyme abundance. These effects are spatially concentrated in hypoperfused tumor regions and fibrotic niches.

Chemotherapy, radiotherapy and targeted therapy introduce a second wave of inflammatory selection. Acute DNA damage may release nucleic acids and activate cGAS–STING, but dying cells also induce wound-healing cytokines, macrophage recruitment and fibroblast activation. Surviving tumor cells can enter slow-cycling persister states supported by enhancer rewiring and altered histone marks. Thus, treatment can transiently increase immunogenicity while simultaneously building a relapse-supportive niche. **Figure 1** summarizes these inputs in a gastric-cancer-specific conceptual architecture, highlighting representative links from inflammatory triggers through signaling and epigenetic regulation to epithelial and microenvironmental states (29–34). Treatment-induced epigenetic responses can be operationally separated into an acute stress response and a persistent epigenetic-memory state. The acute phase remains closely coupled to ongoing DNA damage and tissue injury, with cGAS–STING, NF-κB and wound-repair signaling promoting transient chromatin accessibility, stress-enhancer activation and inflammatory or immunogenic programs. These changes are expected to diminish as the initiating therapeutic stress is withdrawn and tissue repair resolves. By contrast, persistent epigenetic memory is defined by maintenance after the original stress has waned and is associated with selection of slow-cycling persister cells, stabilized enhancer rewiring and histone states, metabolic adaptation, and reciprocal macrophage–fibroblast repair circuits. This durable state can sustain drug tolerance during minimal residual disease even when the initial treatment-induced inflammatory signal is no longer dominant. The two states therefore imply different therapeutic windows: acute responses may provide an opportunity for timely immune consolidation while tumor immunogenicity is increased, whereas persistent states may require biomarker-guided epigenetic, myeloid or stromal reprogramming before resistance becomes genetically fixed. Because the duration of each state varies with treatment modality, genotype and niche context, serial tissue or liquid-biopsy measurements are more informative than fixed calendar thresholds for identifying the transition between transient stress responses and durable drug-tolerant memory (**Table 1**).

**Figure 1:**
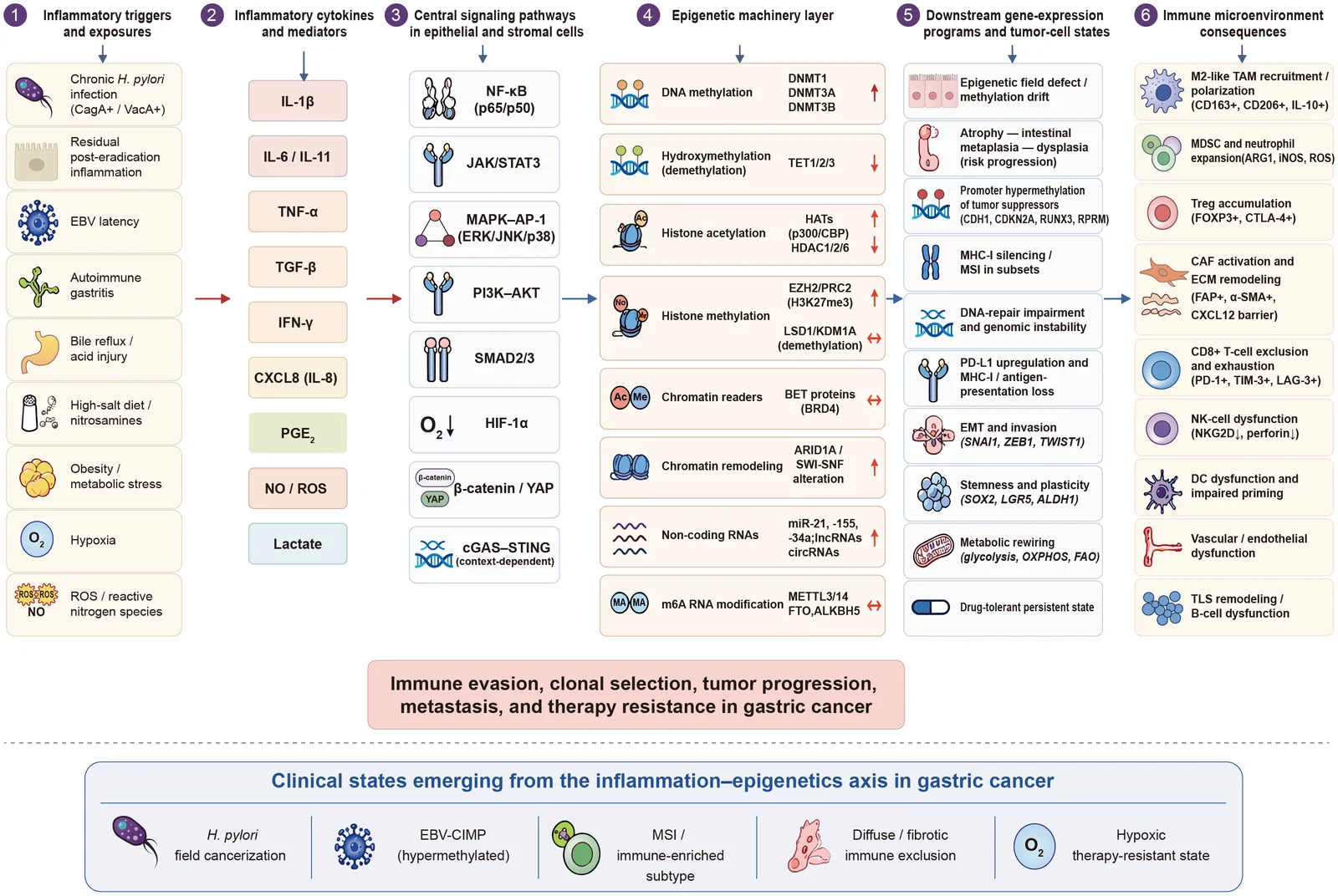
Simplified systems architecture of inflammation-driven epigenetic reprogramming in gastric cancer. Representative inflammatory and metabolic inputs—including *H. pylori*/CagA, persistent post-eradication inflammation, EBV latency, autoimmune gastritis, bile reflux, metabolic stress, hypoxia, and reactive oxygen or nitrogen species—are linked through core NF-κB, JAK–STAT3, MAPK–AP-1, PI3K–AKT, SMAD, HIF-1α, β-catenin/YAP, and cGAS–STING pathways to major epigenetic layers, including DNA methylation and hydroxymethylation, histone regulation, chromatin remodeling, non-coding RNA, and m6A programs. These representative connections converge on field cancerization, metaplasia, defective DNA repair, immune escape, EMT, stemness, and drug-tolerant persistence (29–39).

**Table 1:** Major inflammatory inputs that reprogram the gastric epigenome and tumor microenvironment.

Inflammatory cue/source	Signal integration	Epigenetic execution	Representative consequence	TME phenotype	Potential leverage
H. pylori/CagA and barrier injury	TLR–MyD88, NF-κB, AP-1, SHP2, β-catenin	DNMT induction; CpG methylation drift; enhancer activation; miRNA remodeling	Atrophy–metaplasia field, tumor-suppressor silencing, regenerative proliferation	Neutrophil/macrophage-rich gastritis with long-lived field memory	Eradication; anti-inflammatory interception; methylation-guided surveillance
IL-1β, TNF-α and nitric oxide	IKK–NF-κB; p38/JNK; DNA-damage response	DNMT recruitment; TET/KDM inhibition by oxidative stress; inflammatory super-enhancers	RUNX3 and other gene silencing, DNA repair impairment, stress tolerance	Chronic inflammatory niche that may persist after bacterial clearance	Remove source; redox or cytokine modulation; field-risk monitoring
IL-6/IL-11	JAK1/2–STAT3 with NF-κB cooperation	DNMT/HDAC recruitment; enhancer acetylation; ncRNA loops	Survival, stemness, PD-L1 and suppressive chemokines	Myeloid-rich, checkpoint-high but weakly cytotoxic TME	STAT3/cytokine blockade; chromatin priming plus ICI or myeloid therapy
TGF-β and fibrotic repair	SMAD2/3/4; PI3K–AKT; MAPK	EZH2 and HDAC recruitment; epithelial-gene closure; CAF memory	EMT, diffuse invasion, collagen deposition and cytotoxic-cell exclusion	Fibrotic immune-excluded ecosystem	TGF-β/CAF targeting; vascular or trafficking restoration; selective epigenetic reset
EBV latency	Viral latent programs; NF-κB/IRF; PI3K signaling	Extreme promoter hypermethylation; viral/host ncRNAs; chromatin remodeling	CDKN2A silencing, altered differentiation, checkpoint activation	Immune-rich but adaptively suppressed tumors in many cases	ICI in immune-competent disease; virus-directed or demethylating strategies under study
MSI-associated inflammation	Mismatch-repair loss; cGAS–STING/IFN pressure	MLH1 promoter methylation in sporadic MSI; immune-editing chromatin	High mutation burden and neoantigens, with possible B2M/HLA escape	T-cell-rich tumors with variable irreversible antigen-presentation loss	Distinguish genetic escape from reversible silencing before epigenetic priming
Hypoxia, lactate and lipid stress	HIF-1/2, mTOR, AMPK, metabolic sensors	Reduced dioxygenase activity; altered acetyl-CoA; histone lactylation-related programs	Glycolysis, angiogenesis, macrophage polarization and T-cell dysfunction	Hypoperfused immune-excluded core	Vascular normalization; metabolic intervention; spatial pharmacodynamics
Treatment-induced damage	Acute: cGAS–STING, TLRs, NF-κB; persistent: STAT3 and wound-healing signaling	Acute: stress-enhancer opening; persistent: stabilized persister chromatin and EV/ncRNA signaling	Transient immunogenicity versus slow-cycling drug-tolerant persistence	Early inflammatory activation followed by a persistent myeloid/CAF repair niche	Exploit the acute immune-activation window; reprogram persistent epigenetic and myeloid/CAF repair states

## 3 Epigenetic layers that encode inflammatory memory

### 3.1 DNA methylation, hydroxymethylation and the gastric field defect

DNA methylation is the best-validated molecular memory of chronic gastric inflammation. H. pylori-positive mucosa can show orders-of-magnitude increases in methylation at selected CpG islands compared with uninfected mucosa. The effect includes protein-coding tumor suppressors, DNA-repair genes and microRNA loci. Candidate field markers have included CDH1, p16/CDKN2A, LOX, HAND1, THBD, RPRM, RASGRF1, RIMS1 and others, although assay performance depends strongly on the sampled region, cell composition and stage of atrophy (3, 5–8, 40, 41).

A useful conceptual distinction is between passenger methylation that reports cumulative exposure and driver methylation that alters cell fitness. Driver lesions may silence apoptosis, differentiation, barrier integrity or DNA repair and therefore cooperate with mutations. Passenger loci can still be valuable biomarkers if they are stable and tightly correlated with the underlying field. Prospective studies are beginning to establish this distinction; for example, high methylation burden in non-neoplastic mucosa after endoscopic resection has been associated with substantially greater risk of metachronous gastric cancer (7, 8).

Hydroxymethylation adds another dimension. TET activity depends on oxygen and α-ketoglutarate and can be inhibited by oxidative or metabolic stress. Loss of 5-hydroxymethylcytosine may therefore reflect both lineage change and reduced active demethylation. Measuring 5mC and 5hmC separately can improve mechanistic interpretation, especially in metaplastic mucosa and hypoxic tumors.

### 3.2 Histone acetylation, methylation and chromatin readers

Inflammatory transcription factors recruit histone acetyltransferases (HATs) and BET readers to accessible enhancers, creating sustained cytokine and survival programs. Histone deacetylase (HDAC)-containing complexes can silence epithelial differentiation, chemokine and antigen-presentation genes. In gastric cancer, HDAC expression patterns have been linked to immune composition and prognosis, but broad HDAC inhibition has a narrow therapeutic window because the same enzymes regulate tumor cells, dendritic cells and effector lymphocytes.

EZH2 deposits H3K27me3 and can lock cells into poorly differentiated or stem-like states. LSD1 removes mono- and dimethyl marks and supports enhancer selection, lineage plasticity and stress adaptation. Preclinical studies in cancer models support dependencies on LSD1 and other chromatin regulators, including combinations with chemotherapy or immune priming, but the compartmental effects require direct evaluation (42). BET proteins stabilize transcription at acetylated inflammatory and oncogenic enhancers; their inhibition may collapse IL-6/NF-κB circuitry while also reducing desired lymphocyte activation. Dose and schedule are therefore mechanistic variables, not merely tolerability parameters (33, 34, 37–39).

### 3.3 Chromatin remodeling, enhancer topology and lineage identity

SWI/SNF alterations, particularly ARID1A loss, are frequent in EBV-positive and other gastric cancers. These mutations change nucleosome positioning, enhancer accessibility and immune signaling and may create dependencies on EZH2 or other compensatory regulators. Even without mutation, chronic inflammation can redistribute remodelers and establish metaplasia-associated enhancer landscapes. This is important because intestinal metaplasia is not simply a change in marker expression; it is a stabilized lineage state with altered regulatory topology.

Super-enhancers occupied by NF-κB, STAT3, AP-1, hypoxia-inducible factor (HIF) or lineage factors can maintain cytokine, stemness and anti-apoptotic programs after the original stimulus wanes. Conversely, chromatin compaction can isolate MHC-I, interferon-response or epithelial genes from their regulatory elements. Measurements of global histone marks are therefore insufficient. Mechanistic studies should map locus-resolved accessibility, transcription-factor occupancy and enhancer–promoter contacts in defined cell populations (10, 11, 30–34).

### 3.4 Non-coding RNAs, extracellular vesicles and N6-methyladenosine

MicroRNAs and lncRNAs connect inflammatory signaling to chromatin targeting and post-transcriptional regulation. H. pylori-associated methylation can silence microRNA genes, while NF-κB, STAT3 and HIF induce RNAs that reinforce inflammation, epithelial-to-mesenchymal transition (EMT) or immune escape. Extracellular vesicles (EVs) extend these circuits across cell boundaries: tumor-derived cargo can educate macrophages and fibroblasts, whereas stromal vesicles can sustain tumor-cell plasticity and drug tolerance. Circulating EV RNAs are promising investigational biomarkers, but current evidence is insufficient for routine clinical implementation because source attribution, pre-analytical variability and independent validation remain major limitations.

Non-coding RNA programs also vary across the TCGA molecular subtypes. EBV-positive gastric cancer provides the clearest subtype-specific example, with abundant viral BamHI-A rightward transcript (BART) miRNAs superimposed on host miRNA and lncRNA remodeling; these viral RNAs regulate apoptosis, immune recognition, epithelial plasticity and viral latency. MSI tumors show a distinct RNA-regulatory context shaped by mismatch-repair deficiency, hypermutation and interferon-rich immune pressure, whereas GS/diffuse tumors are more strongly associated with ncRNA programs linked to epithelial–mesenchymal transition, adhesion loss and lineage plasticity. CIN tumors display greater ncRNA heterogeneity in parallel with receptor-tyrosine-kinase signaling, copy-number alterations and cell-cycle stress. Importantly, many reported subtype-associated miRNA and lncRNA signatures derive from retrospective bulk-transcriptomic datasets and may partly reflect differences in tumor purity, immune infiltration and stromal composition. Their clinical use therefore requires independent validation and, where possible, cell-resolved or spatial confirmation.

m6A writers, erasers and readers regulate transcript stability and translation during rapid state transitions. In gastric cancer, m6A circuitry intersects with stemness, immune signaling and chemotherapy response. Because RNA modification changes protein output before chromatin states fully stabilize, it may be especially relevant to early persister formation. Therapeutic targeting is promising but demands lineage-specific pharmacology to avoid impairing normal hematopoiesis and mucosal repair (33–35, 37–39). These epigenetic layers are summarized in **Table 2**.

**Table 2:** Epigenetic layers that encode inflammatory memory in gastric cancer.

Epigenetic layer	Inflammatory linkage	Representative gastric examples	Functional output	Best mechanistic readout	Translational opportunity
Promoter/enhancer DNA methylation	H. pylori-driven cytokines, NO/ROS, DNMT induction	CDH1, CDKN2A, RUNX3, RPRM, RASGRF1, RIMS1; miRNA loci	Field cancerization, differentiation and repair-gene silencing	Cell-type-resolved bisulfite sequencing; longitudinal mucosal sampling	Risk stratification, chemoprevention trials, DNMT priming; phase I azacitidine priming has been evaluated in resectable gastric/esophageal adenocarcinoma (43)
5hmC and active demethylation	Hypoxia and altered α-ketoglutarate/TET activity	Global and locus-specific 5hmC loss in atrophy or tumor states	More fixed methylation and lineage restriction	Oxidative bisulfite or enzymatic 5hmC assays	Improve field interpretation and response monitoring
Histone acetylation/HDAC balance	NF-κB/STAT3 enhancer activation; HDAC recruitment	Chemokine, MHC-I, epithelial and stress-response loci	Inflammatory enhancer persistence or immune silencing	ChIP-seq/CUT&Tag plus ATAC in sorted cells	Short priming schedules; isoform-selective HDAC strategies; vorinostat plus capecitabine/cisplatin has undergone phase I/II evaluation in advanced gastric cancer (44)
H3K27me3/EZH2	TGF-β, stemness and SWI/SNF compensation	Differentiation, interferon and antigen-presentation repression	Lineage locking, EMT, immune escape	CUT&Tag and perturbation with lineage tracking	EZH2 combinations in selected ARID1A/lineage contexts
LSD1 and enhancer choice	Stress, STAT3/AP-1 and persister formation	Stemness and drug-tolerant chromatin dependencies	Plasticity, metastatic competence and resistance	Perturb-seq; enhancer occupancy; persister assays	LSD1 inhibition with chemotherapy or immune activation
BET readers/super-enhancers	Acetylated NF-κB/STAT3/AP-1 domains	IL-6, MYC and survival circuitry	Cytokine amplification and oncogenic addiction	BRD4 occupancy and nascent transcription	BET blockade with careful immune timing
ncRNAs and EV cargo	Induction or methylation by H. pylori, EBV, hypoxia	miRNAs, EBV RNAs, lncRNA scaffolds and vesicle cargo	Feedback loops and intercellular reprogramming	Small-RNA sequencing with source deconvolution	Liquid biomarkers and cell–cell communication targets
m6A RNA modification	Inflammatory and metabolic regulation of writers/readers	Stemness-, checkpoint- and stress-response transcripts	Rapid translational adaptation	m6A-seq, direct RNA methods and ribosome profiling	Target early state transitions; monitor toxicity

## 4 Tumor-cell reprogramming: from inflamed mucosa to immune escape

### 4.1 Atrophy–metaplasia transition and field selection

The atrophy–metaplasia transition is a lineage reprogramming event rather than a passive marker of damage. Loss of acid-secreting and chief-cell programs, expansion of regenerative progenitors and acquisition of intestinal transcriptional networks are accompanied by altered DNA methylation and enhancer accessibility. Repeated inflammatory cycles select glandular clones that tolerate oxidative stress and regenerate efficiently. These clones need not be malignant, but they form a permissive ecological field.

Because field lesions extend beyond visible tumors, surgical or endoscopic removal does not reset risk. Residual mucosa may retain the same methylation burden that facilitated the index lesion. This provides a biological explanation for metachronous cancer after endoscopic resection and supports post-treatment surveillance based on molecular field measurements rather than tumor pathology alone (30–32, 34).

### 4.2 Antigen presentation, interferon adaptation and checkpoint expression

Gastric tumor cells can evade immunity by reducing antigen generation, peptide transport, MHC-I assembly or interferon responsiveness. These defects may be genetic—such as B2M or HLA alterations in hypermutated tumors—or epigenetic through promoter methylation, repressive histone marks and enhancer closure. The distinction is therapeutically decisive: genetically deleted machinery will not be restored by chromatin therapy, whereas epigenetically repressed pathways may reopen.

Inhibition of DNA methyltransferases (DNMTs) or HDACs can derepress endogenous retroelements and interferon-stimulated genes, producing viral mimicry and increased antigen presentation. Yet inflammatory priming also induces PD-L1, adenosine pathways and suppressive chemokines. EBV-positive tumors exemplify this duality: immune infiltration and viral antigens coexist with strong checkpoint adaptation. Productive priming therefore requires both tumor-cell competence and a microenvironment capable of converting interferon activation into cytotoxic control (37–39).

### 4.3 EMT, stemness and diffuse-lineage plasticity

TGF-β, IL-6/STAT3, NF-κB and hypoxia recruit DNMTs, HDACs, EZH2, LSD1 and remodelers to suppress epithelial genes and activate motility, stemness and survival programs. Gastric EMT is best understood as a continuum of hybrid states. Partial EMT can preserve proliferative capacity while increasing invasion and resistance; complete mesenchymal conversion is not required.

The GS/diffuse subtype illustrates the interaction between genetic and epigenetic plasticity. CDH1 and RHOA-pathway alterations change adhesion and cell mechanics, while chromatin programs determine whether cells maintain gastric, metaplastic, basal-like or mesenchymal identities. Therapies that target chromatin may be most effective when the relevant lineage state remains plastic rather than genetically fixed (34, 45).

### 4.4 Drug-tolerant persisters and minimal residual disease

Cytotoxic and targeted therapies eliminate proliferating cells but can leave slow-cycling persisters. These cells frequently depend on reversible enhancer remodeling, histone demethylation, autophagy and altered metabolism. Macrophage and fibroblast repair signals then stabilize the persister niche. The clinical consequence is a period of minimal residual disease in which tumor burden is low but evolutionary potential is high.

A rational intervention should exploit this temporal window. Epigenetic therapy given too early may increase toxicity or protect quiescent cells; given too late, it may encounter genetically consolidated resistance. Serial cell-free DNA (cfDNA), transcriptomic or chromatin-state measurements can identify whether a regimen has induced immunogenic cell death, a myeloid repair rebound or a persistent lineage transition (34, 45).

## 5 Reprogramming of immune, stromal and vascular compartments

### 5.1 Myeloid cells: suppressive enhancers and wound-healing memory

Tumor-associated macrophages (TAMs), myeloid-derived suppressor cells (MDSCs) and neutrophils are major interpreters of chronic gastric inflammation. Tumor-derived CSF1, CCL2, CXCL8, IL-6, IL-10, TGF-β, lactate and EV cargo recruit and educate myeloid cells. These populations do not fit a binary M1/M2 model. Single-cell studies reveal multiple states associated with inflammatory sensing, lipid metabolism, matrix remodeling, angiogenesis, antigen presentation and T-cell suppression (46).

Suppressive myeloid states are maintained by accessible enhancers, acetylation and DNA methylation patterns. They inhibit immunity through ARG1, ROS, IL-10, TGF-β, checkpoint ligands and exclusionary chemokines. Chromatin priming of tumor cells may paradoxically intensify myeloid recruitment, so combinations with CSF1R, CCR2, CXCR2 or IL-6/STAT3 strategies are most rational in myeloid-dominant tumors and should be guided by serial myeloid-state readouts (14, 47–50).

### 5.2 Dendritic cells and the failure of cross-presentation

Dendritic cells determine whether tumor antigen release becomes productive T-cell priming. Chronic IL-10, TGF-β, VEGF, lipid overload and hypoxia can reduce antigen processing, co-stimulation and IL-12. Some defects are maintained by HDAC activity and closed antigen-presentation chromatin. Numerical presence of dendritic cells therefore does not imply function.

Restoration requires more than tumor-cell death. It may require innate-sensing activation, metabolic rescue and relief from suppressive cytokines. Spatial location matters: dendritic-cell niches near vascular entry points and stem-like T cells are likely more consequential than bulk abundance (49–52).

### 5.3 T cells, natural killer cells and epigenetic exhaustion

Chronic antigen and inflammatory signaling establish T-cell exhaustion through TOX, NR4A, BATF-family factors and durable enhancer accessibility. Early dysfunctional states retain proliferative potential, whereas terminal exhaustion is difficult to reverse. Checkpoint blockade can reactivate transcription but does not fully erase the exhaustion epigenome. This explains why the abundance of stem-like tumor-reactive T cells and the quality of tertiary lymphoid structures (TLS) can be more informative than total CD8 density.

NK cells are suppressed by TGF-β, hypoxia, lactate and adenosine and may undergo chromatin closure at cytotoxic genes. NK-mediated control is particularly relevant when tumor MHC-I is low. Clinical studies should therefore profile both T and NK compartments and distinguish antigen-presentation loss that favors NK recognition from broad immune exclusion that disables both lineages (53–56).

### 5.4 Cancer-associated fibroblasts and endothelium: structural memory of inflammation

Gastric CAFs occupy inflammatory, myofibroblastic, CTHRC1-positive and other context-dependent states. IL-1/NF-κB favors cytokine-rich inflammatory programs, whereas TGF-β favors matrix-producing states. Single-cell analyses have associated specific activated fibroblast states with poor outcome and immunotherapy resistance (46). Epigenetic memory and reciprocal signaling between CAF subsets create stable spatial niches rather than a homogeneous stroma.

Endothelial cells integrate VEGF, hypoxia and inflammatory signals and can reduce ICAM1, VCAM1 and chemokines needed for lymphocyte entry. Abnormal vessels also limit drug delivery and generate metabolic stress. Vascular normalization should therefore be viewed as a way to reset the inflammatory–epigenetic ecosystem, not only as an anti-angiogenic intervention. Recent spatial studies of gastric cancer support the importance of fibroblast–myeloid–vascular neighborhoods in primary resistance (16, 19). **Figure 2** resolves the ecosystem by cellular compartment and maps the dominant resistance bottlenecks to representative intervention points (14, 47–50, 57, 58). The corresponding cell-resolved states are summarized in **Table 3**.

**Figure 2:**
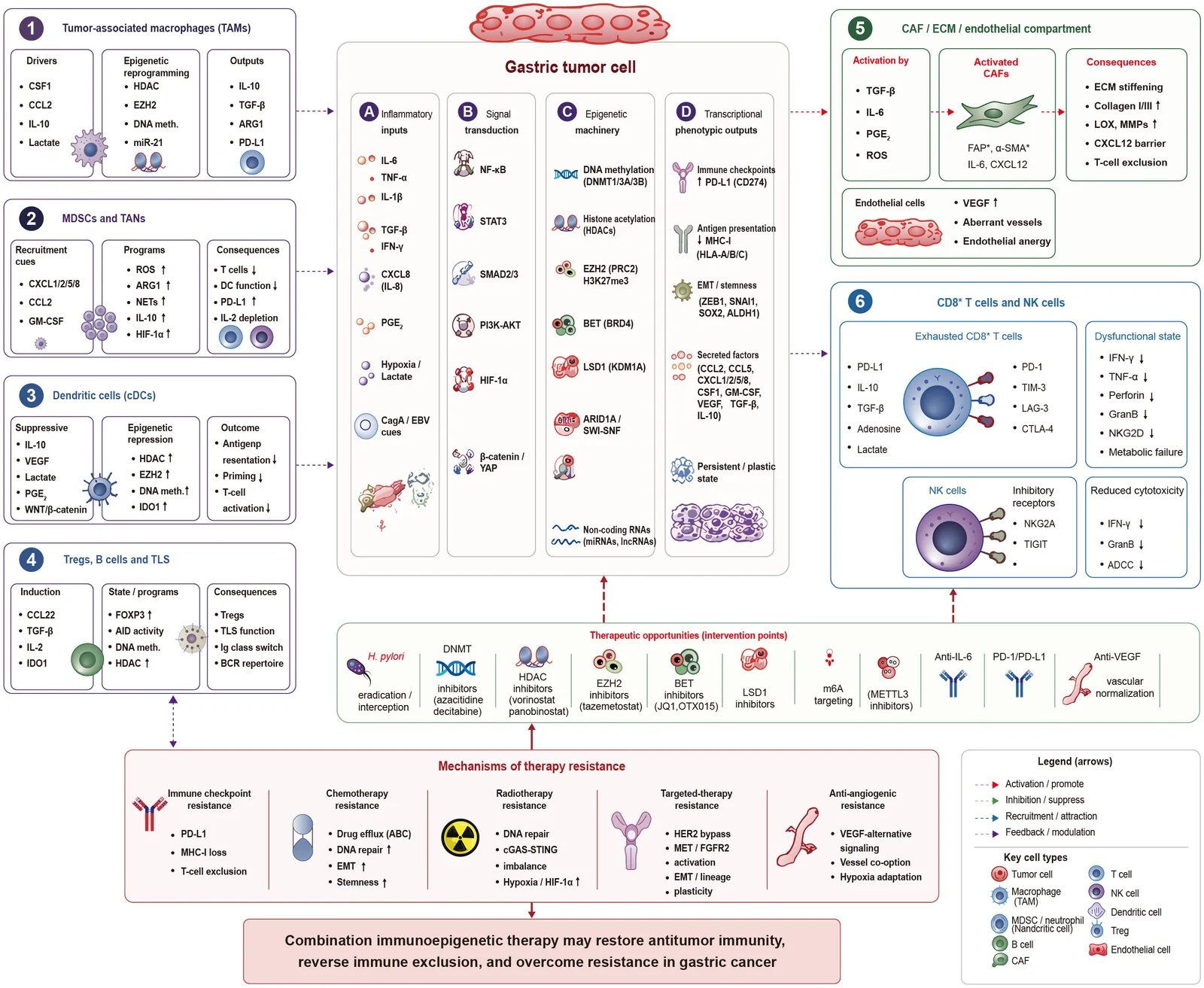
Simplified cell-resolved epigenetic ecosystem, resistance mechanisms, and intervention points in gastric cancer. The schematic organizes major inflammatory inputs, signaling and epigenetic machinery in malignant cells alongside representative myeloid, dendritic, lymphoid, fibroblast, and endothelial states. These compartment-specific programs converge on antigen-presentation loss, checkpoint adaptation, EMT and stemness, persister states, immune exclusion, and therapy resistance. Representative intervention points are shown to emphasize state-matched rather than indiscriminate combination therapy (14–22, 33, 37–39, 45, 47–56).

**Table 3:** Cell-resolved epigenetic states within the gastric tumor microenvironment.

TME compartment	Inflammatory inputs	Epigenetic state/mechanism	Functional output	Useful readouts	Therapeutic implication
Malignant epithelial cells	H. pylori/EBV history; IL-6, TNF, TGF-β, IFN, hypoxia	DNA methylation; HDAC/EZH2 repression; enhancer rewiring; ncRNA/m6A	Antigen loss, PD-L1, EMT, stemness, persisters	Methylome, ATAC, MHC-I/IFN scores, lineage markers	Match priming to intact genetic competence and immune context
TAMs/MDSCs/neutrophils	CSF1, CCL2/CXCL8, IL-6/10, TGF-β, lactate, lipids	Suppressive enhancer accessibility; acetylation and methylation remodeling	ARG1/ROS, IL-10/TGF-β, angiogenesis, T-cell suppression	Single-cell RNA/ATAC, spatial myeloid neighborhoods, plasma chemokines	Combine chromatin therapy with recruitment/survival blockade in myeloid-dominant states
Dendritic cells	IL-10, VEGF, TGF-β, lipid overload, hypoxia	HDAC-linked repression and reduced accessible antigen-processing loci	Weak cross-presentation, low IL-12 and poor priming	DC subset abundance, co-stimulation, IL-12, antigen uptake	Add innate priming and relieve suppressive cytokines
CD8 T cells	Chronic antigen, IFN, TGF-β, adenosine and nutrient stress	Accessible exhaustion enhancers; TOX/NR4A programs	Checkpoint expression, reduced cytokines and killing	TCR clonality; stem-like versus terminal markers; ATAC	ICI is most effective before terminal epigenetic fixation
NK cells	TGF-β, hypoxia, lactate and adenosine	Closure of cytotoxic genes and metabolic reprogramming	Reduced perforin, IFN-γ and activating receptors	NK-state profiling, ligand–receptor maps, cytotoxicity assays	Restore metabolism/activation, especially in MHC-I-low tumors
CAFs	TGF-β, IL-1, IL-6 and mechanical stress	Stable inflammatory or myofibroblastic enhancer landscapes	Collagen/CXCL12, cytokines, invasion and immune exclusion	Spatial CAF states, matrix architecture, TGF-β activity	Target dominant CAF program rather than indiscriminate depletion
Endothelium	VEGF, hypoxia and TNF-family signals	Repression of adhesion and trafficking programs	Low lymphocyte entry, abnormal perfusion and drug delivery	ICAM1/VCAM1, vessel maturity, immune entry points	Vascular normalization can enable immune and epigenetic therapy
Tregs/B cells/TLS	TGF-β, IL-2, antigen and local metabolites	Stable Treg lineage or context-specific B/TLS programs	Suppression or productive local immunity	Spatial proximity, clonality, TLS maturation and antibody programs	Avoid expanding suppressive niches; preserve productive TLS

## 6 Gastric molecular and etiologic archetypes

### 6.1 *H. pylori*-associated intestinal-type field cancerization

This archetype begins with chronic active gastritis and progresses through atrophy and intestinal metaplasia. The defining epigenetic feature is a broad mucosal field rather than a tumor-confined lesion. Methylation burden reflects cumulative inflammatory exposure and residual risk after eradication. Tumors arising in this field can fall into several TCGA classes, so etiology and molecular subtype should be recorded separately.

H. pylori eradication reduces incident and metachronous gastric cancer but does not eliminate risk once atrophy, metaplasia, or a high methylation burden is established (59, 60). Post-eradication surveillance should therefore be stratified by residual mucosal risk rather than by infection status alone.

### 6.2 EBV-positive hypermethylated and immune-adapted cancer

EBV-positive cancer combines extreme CpG island methylation with immune visibility. Viral antigens and interferon-rich inflammation can attract lymphocytes, yet PD-L1/PD-L2 expression, JAK2 activation and epigenetic antigen adaptation blunt effective control. Viral and host epigenomes are coupled: DNA methylation maintains latency, while viral products shape host transcription and chromatin (9, 25–28).

### 6.3 MSI and MLH1-linked CIMP

Sporadic MSI gastric cancers frequently arise through MLH1 promoter methylation and carry high mutation burdens. Their immune phenotype can be favorable because of abundant neoantigens, but ongoing immune editing can select B2M or HLA defects that are genetically irreversible. Epigenetic priming should therefore not be assumed necessary in every MSI tumor; the dominant bottleneck may already be checkpoint inhibition, or it may be structural antigen-presentation loss.

MSI also illustrates why the label CIMP is insufficient. EBV-CIMP and MLH1-linked CIMP involve different loci, viral context, mutation spectra and treatment hypotheses. Biomarker panels should report subtype-specific methylation patterns rather than a single hypermethylation score.

### 6.4 Genomically stable/diffuse and chromosomally unstable states

GS/diffuse tumors are enriched for CDH1, RHOA and CLDN18–ARHGAP alterations and often display infiltrative growth, lineage plasticity and stromal dependence. Their inflammatory–epigenetic bottleneck may be TGF-β/CAF exclusion rather than antigenic silence. Chromatin targeting is most plausible when it reverses a defined lineage or persister state and is combined with trafficking or stromal intervention.

CIN tumors carry aneuploidy and receptor-tyrosine-kinase amplifications and may arise in inflamed or metaplastic fields. Targeted therapy and chemotherapy create strong treatment-induced inflammation and persister selection. Epigenetic interventions should therefore be integrated with the specific oncogenic driver and timed around response, rather than applied as pan-CIN therapy (61–63).

Across these etiologic and molecular contexts, gastric carcinogenesis can be viewed as a temporal continuum in which chronic infection or sterile injury establishes epigenetic field changes before dysplasia, whereas invasion and treatment select more stable chromatin and RNA-regulatory states. This evolution also creates distinct biomarker windows for mucosal-field assessment, plasma or stool methylated DNA, spatial tissue profiling and minimal-residual-disease monitoring (3–8, 10–13, 64–67) (**Table 4**).

**Table 4:** Inflammation–epigenetics archetypes across gastric cancer molecular contexts.

Archetype	Inflammatory setting	Dominant epigenetic process	Immune/stromal problem	Biomarker opportunity	Therapeutic priority
H. pylori field/intestinal pathway	Chronic active gastritis, atrophy and metaplasia	Multifocal methylation drift, 5hmC loss and metaplastic enhancer stabilization	Persistent field plus variable tumor immune state	Non-neoplastic mucosal methylation; stool/plasma methylated DNA	Eradication, molecular risk stratification and interception
EBV-positive	Viral latency with lymphoid-rich inflammation	Extreme EBV-CIMP, viral/host ncRNAs and chromatin remodeling	Immune-visible but checkpoint-adapted; variable T-cell competence	EBV status, methylation pattern, PD-L1/T-cell spatial state	ICI when immune competent; demethylating or viral strategies in trials
MSI/gastric CIMP	MLH1 methylation and hypermutation	MLH1-linked CIMP plus immune-editing events	T-cell-rich but possible B2M/HLA genetic escape	MSI/MMR, methylation and antigen-presentation genotype	Checkpoint therapy; avoid futile priming when machinery is deleted
GS/diffuse	Adhesion and RHO-pathway lesions with TGF-β-rich stroma	Lineage plasticity, EMT and stromal enhancer memory	CAF matrix, diffuse invasion and immune exclusion	Lineage markers, CLDN18/CDH1/RHOA status, spatial CAF maps	State reversal plus stromal/trafficking intervention
CIN/RTK-driven	Metaplastic field plus oncogenic and treatment stress	Aneuploidy-associated chromatin stress and persister remodeling	Heterogeneous immune context, hypoxia and therapy-induced repair	Driver genotype, cfDNA dynamics, post-treatment spatial state	Targeted/cytotoxic backbone with time-aware epigenetic or immune combinations

## 7 Therapeutic frontiers

### 7.1 Remove the sustaining inflammatory driver: eradication and interception

H. pylori eradication is the most mature causal intervention on the gastric inflammation–epigenetics axis. Randomized trials show reduced gastric-cancer incidence after eradication, including reduced metachronous cancer after endoscopic resection, and long-term follow-up supports durable benefit (59, 60). The benefit is greatest before advanced atrophy, but even later eradication removes ongoing inflammatory selection.

Eradication is not equivalent to epigenetic reset. Residual methylation, established metaplasia and clonal alterations can persist. A next-generation prevention strategy should therefore pair eradication with a post-eradication molecular field assessment. Patients with low residual field burden may return to standard surveillance, whereas those with high burden could undergo intensified endoscopy, dietary/metabolic intervention or carefully designed chemoprevention trials.

### 7.2 DNMT inhibition as antigenic and differentiation priming

DNMT inhibitors can reactivate tumor suppressors, endogenous retroelements, interferon pathways and MHC-I genes. In EBV-positive disease they may also perturb viral latency and restore differentiation-related programs. The desired effect is reprogramming rather than cytotoxicity, favoring low-dose or intermittent schedules followed by immune activation after sufficient chromatin opening.

Response requires intact downstream sensing. Tumors with B2M deletion, HLA loss or terminally exhausted lymphocytes are unlikely to benefit from antigenic priming alone. DNMT inhibition can also alter hematopoietic and myeloid cells. Pharmacodynamic studies should measure tumor-cell methylation and interferon response, circulating cytokines, myeloid recruitment and T-cell differentiation rather than relying on global demethylation (33, 37–39).

### 7.3 HDAC, EZH2, LSD1 and BET targeting

HDAC inhibitors can restore chemokines and antigen-presentation genes and sensitize gastric cancer cells to cytotoxic therapy, but broad inhibition may impair lymphocyte function. Short priming followed by washout and checkpoint therapy is a testable sequence when tumor-cell chromatin changes persist longer than systemic immune suppression.

EZH2 inhibition may relieve H3K27me3-mediated differentiation and immune silencing, particularly in tumors with SWI/SNF dysfunction or lineage locking. LSD1 inhibition has shown preclinical antitumor activity in cancer models and may disrupt stemness, lineage plasticity or persister programs (42). BET inhibition can collapse inflammatory super-enhancers but may suppress beneficial T-cell activation. These drugs should be selected using target occupancy and cell-state biomarkers, not only expression of the enzyme (35, 37, 38).

### 7.4 Myeloid, stromal and vascular remodeling

When the dominant bottleneck is myeloid suppression, epigenetic priming should be paired with blockade of recruitment, survival or suppressive signaling. Candidate approaches include CSF1R, CCR2, CXCR2, IL-6/STAT3 and adenosine pathways. The key endpoint is replacement of suppressive myeloid states with antigen-presenting or inflammatory states that support T-cell expansion, not simply reduction in macrophage numbers.

Fibrotic exclusion requires different logic. TGF-β or CAF-directed therapy, matrix remodeling and vascular normalization can open trafficking routes and improve drug delivery. Anti-angiogenic therapy may transiently normalize vessels and has clinical synergy with immunotherapy in selected gastric-cancer settings. Spatial biopsies should verify that lymphocytes enter tumor nests rather than accumulating at the stromal margin (47–52).

### 7.5 Immune checkpoint blockade and combination sequencing

Checkpoint blockade is now integrated into first-line treatment for advanced gastric and gastroesophageal adenocarcinoma in biomarker-defined populations. Trials such as CheckMate 649 and KEYNOTE-859 established survival benefit for PD-1 blockade plus chemotherapy, and long-term follow-up confirms durable benefit in a subset (68–70). Yet most patients do not achieve lasting control, underscoring the need to identify whether failure reflects antigenic silence, myeloid suppression, fibrotic exclusion or terminal lymphocyte dysfunction.

The inflammation–epigenetics framework favors sequential rather than indiscriminate combinations. Chromatin priming can precede immune checkpoint inhibitor (ICI) therapy when antigen-presentation pathways are intact but closed. Myeloid or vascular remodeling may need to precede ICI when immune cells cannot access or function within tumor nests. In a T-cell-rich EBV or MSI tumor, direct checkpoint therapy may be preferable to broad epigenetic manipulation (51, 52, 62, 63, 68, 70).

### 7.6 State-by-time matching and pharmacodynamic windows

An epigenetic state is reversible only under specific conditions. Reversal depends on the initiating inflammatory circuit, genetic lesions, metabolic cofactors, differentiation stage and niche structure. A pragmatic sequence is: remove the sustaining inflammatory source; reset the dominant chromatin bottleneck; create a permissive trafficking and immune window; then consolidate with checkpoint, cytotoxic, targeted or differentiation therapy (**Figure 3**). Serial tissue and liquid measurements should determine when each transition has occurred. Dose and treatment duration are also critical determinants of the immunologic effect of epigenetic therapy. Low-dose or short-course DNMT and HDAC inhibition may favor tumor-cell epigenetic reprogramming, including restoration of interferon signaling, chemokine expression and antigen-presentation programs, while limiting prolonged effects on immune cells. In contrast, higher or sustained exposure may impair effector T-cell or NK-cell function, induce cytopenia, or promote compensatory suppressive responses, thereby narrowing or reversing the desired immune-priming window. Therefore, epigenetic agents used for immune priming should be scheduled according to pharmacodynamic target engagement rather than maximum tolerated dose alone, with treatment withdrawal or washout before immune consolidation when appropriate (**Table 5**).

**Figure 3:**
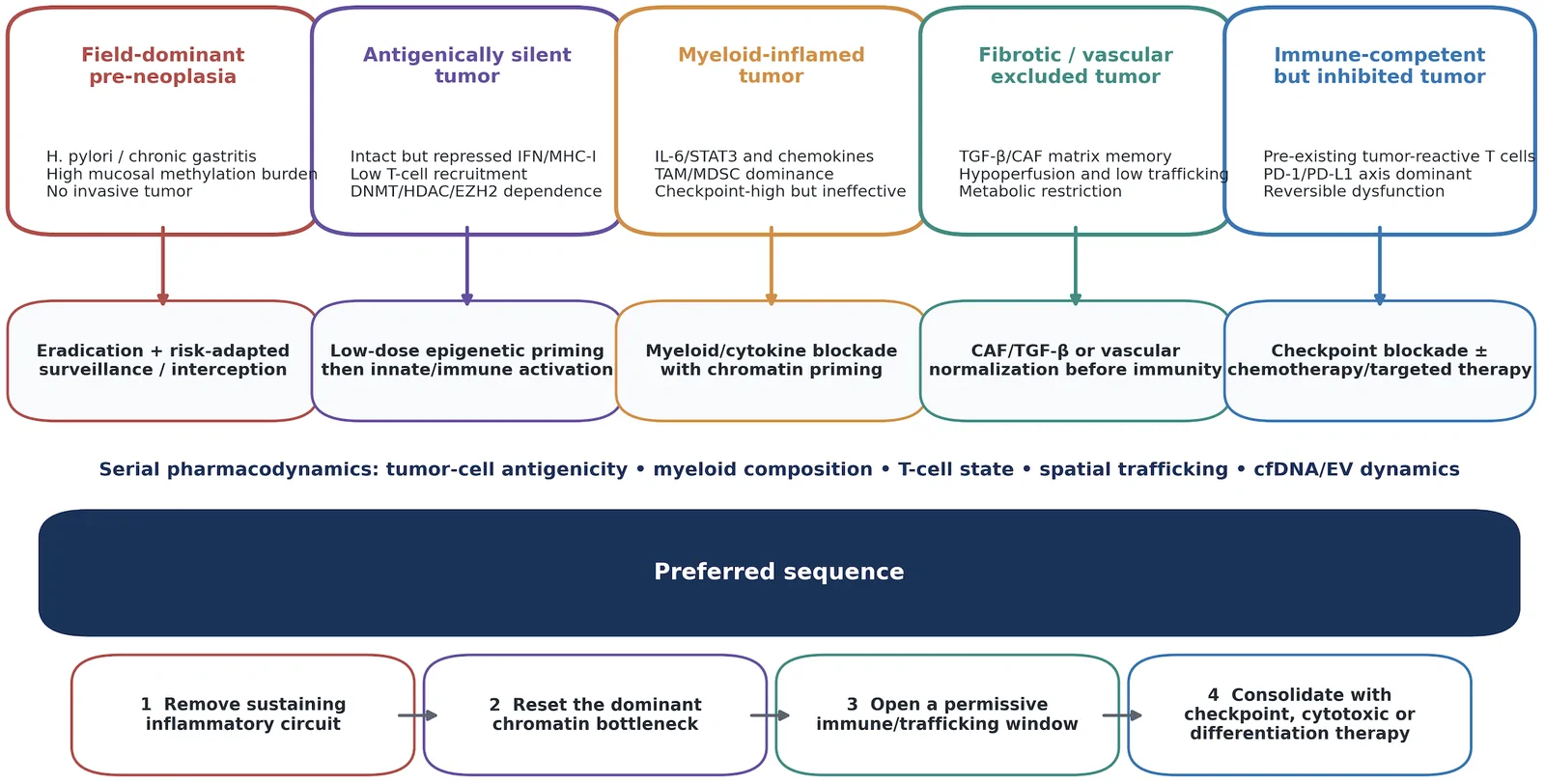
State- and time-matched therapeutic framework. Intervention is selected according to the dominant state—field-dominant pre-neoplasia, an antigenically silent tumor, a myeloid-inflamed tumor, a fibrotic/vascular-excluded tumor, or an immune-competent but inhibited tumor—and aligned with the corresponding interception, epigenetic priming, myeloid/stromal remodeling, or consolidation strategy. Serial pharmacodynamic measurements are used to confirm target engagement and guide progression through the preferred treatment sequence (33, 35, 37–39, 45, 51, 52).

**Table 5:** Mechanism-based therapeutic strategies for the gastric inflammation–epigenetics axis.

Strategy	Biological state selected	Mechanistic objective	Rational partner/sequence	Key pharmacodynamics	Main risk
H. pylori eradication	Active infection or persistent post-treatment inflammation	Remove initiating cytokine/ROS circuit and reduce new methylation	Eradicate first; then reassess field burden and histology	Eradication confirmation, inflammation, mucosal methylation and metaplasia	Residual field risk falsely assumed to be eliminated
Low-dose DNMT inhibition	Intact but methylation-silenced IFN/MHC-I or differentiation programs	Viral mimicry, antigen recovery and tumor-suppressor reactivation	Prime, allow immune recovery/washout, then ICI or innate activation	Locus methylation, ERV/IFN induction, MHC-I, myeloid recruitment	Cytopenia, myeloid rebound or activation without competent immunity
HDAC inhibition	Closed chemokine/antigen loci or chemotherapy tolerance	Increase acetylation and reopen immune/differentiation genes	Short priming before chemotherapy or ICI; isoform-selective where possible	Histone acetylation, CXCL9/10, MHC-I, lymphocyte function	Broad immune suppression and narrow therapeutic window
EZH2 inhibition	H3K27me3-dependent lineage locking; selected SWI/SNF contexts	Release differentiation and interferon programs	Combine with lineage therapy or ICI after target confirmation	H3K27me3 loss, lineage shift, antigenicity	Opposite effects across tumor, Treg and effector compartments
LSD1 inhibition	Stem-like, EMT or drug-tolerant persister state	Disrupt enhancer selection and restore inflammatory sensing	Combine with chemotherapy or immune activation in time-defined window	Enhancer occupancy, persister frequency, lineage markers	Hematopoietic toxicity and context-dependent plasticity
BET inhibition	NF-κB/STAT3 super-enhancer addiction and cytokine amplification	Collapse oncogenic/inflammatory transcriptional hubs	Short exposure plus myeloid or targeted therapy	BRD4 occupancy, nascent transcription, cytokine profile	Suppression of productive T-cell activation
Myeloid/cytokine blockade	TAM/MDSC-dominant, checkpoint-high inflammation	Remove suppressive repair and recruitment circuits	Pair with antigenic priming or ICI	Myeloid state transitions, ARG1/IL-10, T-cell expansion	Compensatory recruitment and infection risk
CAF/TGF-β/vascular remodelling	Fibrotic, hypoperfused immune exclusion	Restore trafficking, perfusion and drug delivery	Remodel niche before or during immune therapy	Matrix architecture, vessel maturity, intratumoral T cells	Loss of restraining stroma or vascular toxicity
PD-1/PD-L1 blockade	Immune-competent but inhibited tumor; many MSI/EBV cases	Release adaptive checkpoint inhibition	Use directly when T-cell competence is present; add priming only for a defined deficit	Stem-like CD8 expansion, ctDNA decline, spatial tumor entry	Immune toxicity and failure in terminal/excluded states

## 8 Biomarkers for interception, stratification and adaptive monitoring

### 8.1 Tissue methylation field markers

Non-neoplastic gastric mucosa is uniquely informative because it records past inflammatory exposure. Methylation of selected loci measured after H. pylori eradication or endoscopic tumor removal can stratify metachronous risk. Recent prospective evidence supports RIMS1 methylation as a risk marker, with individuals in the highest methylation category showing substantially increased future cancer risk. Such assays should be standardized for biopsy site, glandular composition and active inflammation.

Field assays must be interpreted differently from tumor assays. A strong field signal may be clinically useful even when it is not tumor specific; its purpose is to measure the carcinogenic substrate. Validation therefore requires disease-matched controls with gastritis, atrophy and metaplasia, not only healthy controls (3–8, 30–32, 71).

### 8.2 Circulating and stool methylated DNA

cfDNA methylation can support detection, response assessment and minimal-residual-disease monitoring. Recent gastric-cancer studies have developed methylome or targeted panels with promising discrimination, including multi-DMR models, digital-PCR assays and panels containing genes such as GHR, GLRB, GATM, RPRM, SPG20, FBN1, SDC2, TFPI2 and SEPT9. The central unresolved problem is stage I sensitivity at clinically acceptable specificity.

Inflammation is an important confounder. Gastritis and tissue injury release non-malignant DNA and may change methylation in the source tissue. Models should therefore include active H. pylori gastritis, post-eradication atrophy and benign ulcer disease as controls. Fragment size, tissue-of-origin methylation and mutation data can be integrated to distinguish tumor-derived cfDNA from field or leukocyte signals. Stool DNA is especially attractive for gastric and upper-GI field detection when combined with organ-of-origin features, but pre-analytic variability is substantial (64–67).

### 8.3 Circulating RNAs, EVs and immune-cell chromatin

Circulating miRNAs and EV cargo can report active cell–cell communication rather than tumor burden alone. A panel enriched for tumor-derived and myeloid-derived RNAs could identify the transition from an inflammatory field to an immune-suppressive tumor ecosystem. Source deconvolution, hemolysis control and standardized EV isolation are mandatory. Diagnostic miRNA and EV-RNA panels require rigorous statistical validation before clinical application. Development cohorts should include disease-matched controls and standardized serum or plasma processing, with diagnostic performance reported using receiver-operating-characteristic (ROC) curves, area under the curve (AUC), sensitivity, specificity and confidence intervals at prespecified thresholds. Group comparisons should use appropriate parametric or non-parametric tests according to data distribution; ANOVA may be applicable for comparisons across multiple groups but is not sufficient alone for biomarker validation. Multivariable models should undergo internal resampling or cross-validation, followed by testing in an independent external cohort. Public datasets such as GEO or TCGA can support discovery and orthogonal validation when compatible specimens and platforms are available, but they should not substitute for prospective validation of blood-based assays.

Peripheral immune-cell chromatin may provide an orthogonal biomarker. Exhaustion-associated T-cell accessibility, suppressive myeloid enhancers and interferon-response states can be measured longitudinally without repeated tumor biopsy. These signatures should be paired with tumor or spatial data because systemic inflammation may not reflect the intratumoral bottleneck (10–14).

### 8.4 Spatial and single-cell biomarkers

Single-cell RNA sequencing and assay for transposase-accessible chromatin (ATAC) profiling can identify malignant lineage states, activated fibroblasts, suppressive myeloid populations and stem-like versus terminal T cells. Spatial transcriptomics or multiplex imaging then determines whether these states interact within the same niche. Gastric-cancer datasets have highlighted CTHRC1-positive fibroblasts, angiogenic stromal populations, suppressive myeloid cells and regulatory T cells as components of poor-prognosis ecosystems (16, 19, 46).

A clinically useful spatial biomarker should answer a treatment question. Examples include: are antigen-presenting dendritic cells adjacent to stem-like CD8 cells; do lymphocytes enter tumor nests or stop at a collagen-rich margin; is PD-L1 expressed at an inflamed invasive front or diffusely in tumor cells without cytotoxic pressure; and does therapy convert a myeloid-rich niche into a permissive one (10–14)?

### 8.5 Validation principles

Multi-omic biomarker development is vulnerable to information leakage. Feature selection, normalization, batch correction and threshold optimization must occur within training folds; the final model must be locked before external testing. Calibration, decision-curve analysis and prevalence-aware predictive values are as important as area under the curve.

Longitudinal endpoints require time-aware analysis. Samples collected after occult recurrence should not be used as baseline predictors, and repeated measures from one patient must remain in the same data split. External cohorts should differ in geography, endoscopy practice and H. pylori prevalence to establish transportability. Biomarker opportunities are summarized in **Table 6**.

**Table 6:** Biomarker opportunities across prevention, diagnosis and treatment monitoring.

Biomarker class	Biological question	Specimen/platform	Strength	Critical confounder	Validation requirement
Mucosal methylation field score	How much carcinogenic memory remains after infection or tumor removal?	Standardized non-neoplastic biopsies; targeted bisulfite or digital PCR	Directly measures at-risk tissue and can precede cancer	Biopsy site, cell mixture, active inflammation and metaplasia	Prospective metachronous-risk cohorts and site-standardized thresholds
Tumor methylome/5hmC	Is immune or differentiation loss epigenetically reversible?	Tumor tissue; bisulfite/enzymatic assays, methylation arrays or sequencing	Mechanistic target selection	Purity and inability of bulk assays to assign cell type	Perturbation-linked pharmacodynamics and genetic-competence testing
Plasma cfDNA methylation	Is cancer present; is residual disease falling or returning?	Plasma methylome, targeted PCR or bisulfite sequencing	Repeatable and potentially early	Low shedding, leukocyte DNA and gastritis-related tissue injury	Stage-stratified external cohorts, locked model and serial lead-time analysis
Stool methylated DNA	Can a non-invasive assay detect upper-GI field or tumor signals?	Stool DNA methylation ± fragmentomics	Accessible for population screening	Variable transit, degradation and lower-GI sources	Prospective screening cohorts with organ-of-origin adjudication
Circulating miRNA/EV cargo	Which inflammatory cell–cell circuits are active?	Plasma/serum small-RNA and EV profiling	May report communication rather than mass alone	Hemolysis, isolation method and source ambiguity	Standard operating procedures and independent source-validation
Immune-cell chromatin	Are circulating T/myeloid states becoming permissive?	PBMC ATAC/CUT&Tag or targeted accessibility	Longitudinal immune pharmacodynamics	Systemic infection and mismatch with tumor state	Paired tumor/immune measurements and predefined state scores
Spatial tissue signature	Where is the dominant exclusion or immune niche?	Spatial transcriptomics, multiplex imaging, spatial ATAC	Directly resolves neighborhood biology	Sampling area and platform batch effects	Multi-region sampling and outcome-linked external validation
Integrated multi-omic model	Which therapy should be used and when?	Methylation + mutation + RNA + spatial/clinical data	Captures coupled ecosystem states	Overfitting and information leakage	Nested training, locked algorithms, calibration and prospective utility

## 9 Technology and experimental design priorities

### 9.1 Single-cell multi-omics and lineage-resolved perturbation

Single-cell RNA sequencing describes cell states, but inflammation–epigenetics causality requires chromatin and perturbation. Joint RNA–ATAC, single-cell methylation, CUT&Tag and CRISPR perturb-seq can determine whether a cytokine changes cell composition, opens a regulatory element or selects a pre-existing clone. Organoid–immune–fibroblast co-cultures should preserve the relevant etiologic context, including CagA status, EBV infection or metaplastic lineage.

Time is a critical experimental variable. Acute exposure, chronic exposure, withdrawal and re-challenge should be modeled separately. A truly epigenetic memory state persists after removal of the initiating stimulus and changes the response to a later challenge. Studies that measure only the endpoint under continuous cytokine exposure cannot distinguish memory from active signaling (11–13).

### 9.2 Spatial epigenomics and neighborhood causality

Spatial assays should move beyond descriptive maps to causal neighborhood models. Ligand–receptor inference can nominate IL-6, TGF-β, CXCL12 or VEGF circuits, but validation requires perturbing the source cell and measuring downstream chromatin in the recipient cell. Multi-region sampling should include tumor center, invasive margin, metaplastic field and distant mucosa.

A practical clinical design is paired pre-treatment and on-treatment biopsy with plasma sampling. Tissue establishes the spatial bottleneck; plasma measures systemic and tumor-burden dynamics. The combined design can identify whether a treatment failed to hit the target, hit the target without remodeling the ecosystem or remodeled the ecosystem without reducing disease (11–13).

### 9.3 Model systems and prevention trials

Prevention studies require models that reproduce decades of human exposure. H. pylori-infected rodents, human gastric organoids and longitudinal mucosal cohorts each capture different components. No single model reproduces bacterial diversity, immune history, glandular architecture and age-related epigenetic drift. Triangulation is therefore essential.

Interception trials should enrich for biologically high-risk fields rather than histology alone. A feasible design would randomize patients with confirmed eradication but persistently high methylation burden to intensified surveillance or an anti-inflammatory/epigenetic prevention strategy, with serial field measurements and incident neoplasia as endpoints. Safety thresholds must be stricter than in advanced cancer (3–8, 59, 60).

## 10 Discussion

### 10.1 Strength of the causal evidence

The H. pylori methylation field provides the strongest causal chain in this literature. Infection precedes chronic inflammation; experimental suppression of inflammation reduces aberrant methylation; eradication lowers active inflammation and some methylation signals; and residual methylation in non-neoplastic mucosa predicts metachronous cancer (3–8, 59, 60). The main unresolved issue is not whether an epigenetic field exists, but which loci alter epithelial fitness and which merely record cumulative exposure. Prospective studies should therefore combine field measurements with clonal lineage analysis and repeated sampling rather than infer biological function from cross-sectional methylation differences.

### 10.2 Why epigenetic combinations fail

The limited clinical progress of epigenetic therapy in solid tumors is partly explained by inadequate state selection. DNMT or HDAC inhibition can restore interferon signaling and antigen presentation, but the same treatment may induce PD-L1, recruit suppressive myeloid cells, or impair effector lymphocytes (33, 35, 37–39). Priming is unlikely to benefit tumors with irreversible B2M or HLA loss, terminal T-cell exhaustion, or a collagen-rich vascular barrier. Dose and sequence are therefore mechanistic determinants: short, low-intensity priming should be followed only when pharmacodynamic sampling confirms pathway reopening without dominant immune suppression.

### 10.3 Spatial and temporal heterogeneity

Recent single-cell and spatial studies show that gastric cancer contains reproducible but non-uniform immune and stromal neighborhoods. Alternative T-cell exhaustion trajectories, signet-ring-cell-specific states, IGF1-positive fibroblasts, B-cell–tumor crosstalk, and spatially organized resistance to anti-PD-1 plus anti-angiogenic therapy have each been reported (15–20). Peritoneal metastases further display organ-specific stromal and macrophage programs that are not captured by the primary tumor (21). These observations argue against biomarker transfer across anatomical sites or treatment stages without direct validation.

### 10.4 Clinical translation and trial design

A clinically useful trial should enroll patients on the basis of a measurable bottleneck rather than enzyme expression alone. Eligibility might require a preserved antigen-presentation genotype with epigenetic silencing, a myeloid-dominant spatial phenotype, or a persistently methylated post-eradication field. Paired pretreatment and early on-treatment biopsies should assess target engagement in malignant and immune compartments, while plasma methylation or cfDNA should track tumor burden. The investigational epigenetic agent should be discontinued if the intended chromatin change is absent or if an opposing myeloid or stromal program becomes dominant. Such adaptive designs are more informative than fixed combinations tested in molecularly unselected populations.

### 10.5 Limitations and research priorities

Most available data remain observational, and many gastric-cancer studies infer epigenetic state from RNA expression rather than directly measuring chromatin or DNA modification. Cell composition, active gastritis, anatomical site, prior eradication, and treatment exposure are frequent confounders. Priorities include longitudinal post-eradication cohorts, matched primary and metastatic sampling, lineage-resolved methylome and chromatin profiling, and functional perturbation in organoid–immune–stromal models. Newer spatial and single-cell datasets improve resolution but still require independent clinical validation and standardized analytical pipelines (10, 11, 14, 15, 17, 18, 20–22, 72).

## 11 Conclusions

The inflammation–epigenetics axis in gastric cancer is a causal continuum from microbial or sterile injury to field cancerization, malignant plasticity and ecosystem-level immune resistance. H. pylori-associated methylation provides the clearest example of durable inflammatory memory, while EBV-positive and MSI tumors demonstrate mechanistically distinct hypermethylated states. Across subtypes, tumor-cell chromatin interacts with myeloid, lymphocyte, fibroblast and endothelial programs to determine whether inflammation becomes protective or tumor promoting.

Clinical translation will require state-specific and time-aware intervention. The sustaining inflammatory source should be controlled first; the dominant lesion must then be assigned to a defined cellular compartment and shown to be reversible before chromatin-directed treatment is used. Immune, stromal, vascular, or differentiation therapy should be selected according to the residual bottleneck. Biomarker studies must separately quantify active gastritis, non-neoplastic field effects, and tumor-derived signals, with external and longitudinal validation.

A practical route to clinical implementation will require a staged translational pipeline. Candidate biomarkers should first undergo analytical standardization and locked multicenter validation in clinically accessible specimens, followed by conversion into reproducible targeted assays compatible with routine pathology or molecular laboratories. Tissue-based methylation or RNA markers could be incorporated as companion modules alongside established next-generation sequencing (NGS) workflows, whereas circulating biomarkers may be more suitable for longitudinal surveillance and minimal-residual-disease monitoring. Prospective biobanking should link serial blood, non-neoplastic mucosa and tumor tissue with treatment exposure and clinical outcomes. Only biomarkers that demonstrate analytical validity, clinical validity and clinical utility should progress toward commercial diagnostic kits or routine referral pathways.
